# Colonization with multidrug-resistant bacteria among children hospitalized abroad—a study from Finland

**DOI:** 10.1093/jtm/taag003

**Published:** 2026-01-22

**Authors:** Hilda Mäkinen, Mikael Kajova, Tamim Khawaja, Anu Kantele

**Affiliations:** FIMAR, Finnish Multidisciplinary Center of Excellence in Antimicrobial Resistance Research, University of Helsinki, Biomedicum 1, Haartmaninkatu 8, 00029 Helsinki, Finland; Meilahti Vaccine Research Center MeVac, University of Helsinki and Helsinki University Hospital, Biomedicum 1, Haartmaninkatu 8, 00029 Helsinki, Finland; Human Microbiome Research Program, Faculty of Medicine, University of Helsinki, Biomedicum 1, Haartmaninkatu 8, 00029 Helsinki, Finland; FIMAR, Finnish Multidisciplinary Center of Excellence in Antimicrobial Resistance Research, University of Helsinki, Biomedicum 1, Haartmaninkatu 8, 00029 Helsinki, Finland; Meilahti Vaccine Research Center MeVac, University of Helsinki and Helsinki University Hospital, Biomedicum 1, Haartmaninkatu 8, 00029 Helsinki, Finland; Human Microbiome Research Program, Faculty of Medicine, University of Helsinki, Biomedicum 1, Haartmaninkatu 8, 00029 Helsinki, Finland; Department of Infectious Diseases, Inflammation Center, University of Helsinki and Helsinki University Hospital, Haartmaninkatu 4, 00029 Helsinki, Finland; FIMAR, Finnish Multidisciplinary Center of Excellence in Antimicrobial Resistance Research, University of Helsinki, Biomedicum 1, Haartmaninkatu 8, 00029 Helsinki, Finland; Meilahti Vaccine Research Center MeVac, University of Helsinki and Helsinki University Hospital, Biomedicum 1, Haartmaninkatu 8, 00029 Helsinki, Finland; Human Microbiome Research Program, Faculty of Medicine, University of Helsinki, Biomedicum 1, Haartmaninkatu 8, 00029 Helsinki, Finland; Department of Infectious Diseases, Inflammation Center, University of Helsinki and Helsinki University Hospital, Haartmaninkatu 4, 00029 Helsinki, Finland; FIMAR, Finnish Multidisciplinary Center of Excellence in Antimicrobial Resistance Research, University of Helsinki, Biomedicum 1, Haartmaninkatu 8, 00029 Helsinki, Finland; Meilahti Vaccine Research Center MeVac, University of Helsinki and Helsinki University Hospital, Biomedicum 1, Haartmaninkatu 8, 00029 Helsinki, Finland; Human Microbiome Research Program, Faculty of Medicine, University of Helsinki, Biomedicum 1, Haartmaninkatu 8, 00029 Helsinki, Finland; Department of Infectious Diseases, Inflammation Center, University of Helsinki and Helsinki University Hospital, Haartmaninkatu 4, 00029 Helsinki, Finland

**Keywords:** MDR bacteria, MDRO, hospitalization, colonization, children, ESBL, MRSA

## Abstract

**Background:**

International travel contributes to the global spread of antimicrobial resistance: a substantial proportion of travellers visiting low- and middle-income countries (LMICs) are colonized by multidrug-resistant organisms (MDRO), with those hospitalized abroad at a particular risk. In some, colonization leads to symptomatic MDRO infection. Although children are a recognized risk group, research on travel-acquired MDRO among paediatric patients remains limited.

**Methods:**

At our hospital, patients hospitalized abroad within the past 12 months are routinely screened for MDROs upon admission. To assess MDRO colonization among children following hospitalization abroad, we analysed MDRO screening data from paediatric patients at HUS Helsinki University Hospital 2010–2024, and explored associated risk factors.

**Results:**

Among the 459 paediatric patients screened after hospitalization abroad, 158 (34.4%) were colonized with MDROs. The most common MDROs were extended-spectrum β-lactamase-producing Enterobacterales (29.0%) and methicillin-resistant *Staphylococcus aureus* (7.6%). Carbapenemase-producing Enterobacterales were identified in 14 children (3.1%).

Multivariable analysis identified antibiotic use (*P* = 0.002), travel type (*P* < 0.001) and income level of the hospitalization country (*P* < 0.001) as independent risk factors for colonization. The income level gradient was substantial: 87.5% (21/24) of children hospitalized in low-income countries, 68.1% (49/72) in lower-middle-income, 46.6% (55/118) in upper-middle-income and 13.5% (33/245) in high-income countries were colonized with MDROs. Clinical MDRO infection was recorded in five of the 158 (3.2%) MDRO carriers.

**Conclusions:**

MDRO colonization is common among children hospitalized abroad, showing a clear gradient increase with decreasing country income level. Screening and infection control measures are warranted after recent care abroad. Particular focus should be placed on those hospitalized in LMICs, and those with additional risk factors such as visiting friends and relatives travel, foreign residence, or recent antibiotic use.

## Introduction

Globally, young children bear a disproportionate share of antimicrobial resistance (AMR)–attributable mortality,^1,2^ and even in Europe infants remain the most affected age group, with almost 2000 disability-adjusted life-years lost per 100 000 infants.^1,2^ Infections caused by multidrug-resistant organisms (MDRO) are linked to higher mortality, longer hospital stays and increased healthcare costs compared to those caused by antibiotic-susceptible bacteria.^1,3^ In children, treatment options are more limited than in adults due to the smaller number of drugs approved for paediatric use.^4^ Despite this, most studies on MDRO acquisition and infections have focused on adults, while data on paediatric populations remain limited.

The prevalence of multidrug-resistant (MDR) bacteria is highest in low- and middle-income countries (LMICs). Travellers visiting those regions often acquire MDR bacteria and thereby contribute to their global dissemination.^5–9^ As international travel continues to rise, an increasing number of children are also travelling to foreign countries. Many of them are visiting friends and relatives (VFR), a travel pattern that places them at a higher risk of infectious travel-related illnesses.^10^ Risk of acquiring MDROs during travel is particularly high during hospitalization abroad.^7,11–13^ After returning home, children can transmit MDROs to other patients, family members^14,15^ and peers, particularly in daycare settings.^16–18^ Importantly, emerging evidence suggests that colonization may persist longer^15,19^ and, even in low-prevalence settings, colonization rates can be comparatively high among children,^16,20^ potentially amplifying their contribution in MDRO transmission. Yet, studies on MDRO colonization among children hospitalized abroad remain scarce.

## Methods

### Study design

To assess the prevalence and risk factors of MDRO acquisition among paediatric patients hospitalized abroad, we conducted a retrospective cohort study using patient records of children screened at HUS Helsinki University Hospital (HUS) after hospitalization abroad within the previous 12 months. Data were retrieved for all eligible patients screened between January 2010 and December 2024.

### MDRO screening at HUS

Since 2010, the infection control guidelines of HUS have instructed screening of all patients with history of hospitalization abroad within the past 12 months for methicillin-resistant *Staphylococcus aureus* (MRSA) and multidrug-resistant gram-negative bacteria (MRGN), comprising extended-spectrum beta-lactamase-producing Enterobacterales (ESBL-PE), carbapenemase-producing Enterobacterales (CPE), multidrug resistant *Acinetobacter* species (MDRACI) and multidrug resistant *Pseudomonas aeruginosa* (MDRPA). Vancomycin-resistant *Enterococcus* (VRE) screening was included for all patients hospitalized abroad 2010–2016; and since 2016, it was limited to direct interhospital transfers. Minor updates to the screening policy during the study period have been described elsewhere.^11,12,21^

### Patient selection; inclusion and exclusion criteria

Patients screened for both MRSA and MRGN between 1 January 2010 and 31 December 2024 were identified from the HUS Electronic Infection Control database (SAI). Inclusion required MRSA (nose, throat and rectal swabs) and MRGN (rectal swab or stool sample) screening. Additional sample sites (wounds, trachea, catheter sites, catheter urine) were recorded when available. VRE screening results were documented when present but not required for inclusion. Patients were eligible if they were under 18 years and had been hospitalized abroad within the past 12 months, defined as over 24 hours of inpatient care or any invasive procedure abroad. The month of hospitalization had to be recorded for inclusion. Patients with hospitalization in multiple regions (different geographic regions or multiple countries in case of European travel) or documented travelling in multiple regions (different geographic regions) were excluded. Regions were categorized as North America, Central/South America, Sub-Saharan Africa, North Africa/Middle East, Asia and Europe.

### Data collection

We collected data on destinations, travel-related factors, hospitalization, antibiotic use and underlying diseases. Travel types were categorized into three groups: visiting friends and relatives, residence abroad and leisure or other travel. Antibiotic use included both reported use and likely use based on documented bacterial infections or surgeries requiring antibiotic prophylaxis. The Charlson co-morbidity (CCI) index was used to assess underlying conditions. World Bank country income level classification for the year of hospitalization was used to categorize countries (low, lower-middle, upper-middle, high).^22^

### Microbiological methods

All screening samples were analysed at the HUS laboratory HUSLAB using routine protocols, as described previously in detail.^11–13,21^ In brief:

MRSA was detected after overnight enrichment in selective broth (eMRSA, Copan Italia, Brescia, Italy or in-house MRSA broth) and cultured on CHROMagar MRSA (CHROMagar, Paris, France), followed by confirmation by PCR or eazyplex MRSA assay targeting *S. aureus* nuclease and mecA/mecC genes (Amplex).^21^

ESBL-PE and CPE were identified by direct plating on CHROMagar ESBL and CHROMagar KPC or mSuperCARBA, followed by species identification using MALDI-TOF (Vitek-MS, bioMérieux) or VITEK-GN (bioMérieux).^21^ Antimicrobial susceptibility was determined using Clinical and Laboratory Standards Institute (CLSI) guidelines until 2011, and the European Committee on Antimicrobial Susceptibility testing (EUCAST) guidelines thereafter.^23,24^ CPE were confirmed by PCR assays targeting carbapenemase genes (Amplidiag CarbaR+MCR, Mobidiag Ltc, Finland; or Novodiag Carba+ Assay, Hologic, Marlborough, USA).^21,25^

VRE detection included enrichment in Enterococcosel broth (BBL, Cockeysville, MD, USA), culture on CHROMagar-VRE and VanA/VanB gene detection with PCR or easyplex VRE.^21^

MDRACI and MDRPA were screened based on growth on ESBL and *Klebsiella pneumoniae* carbapenemase (KPC) or mSuperCARBA plates followed by species identification on C-390, VITEK-GN, or MALDI-TOF and carbapenemase gene PCR.^21^

MDR isolates of the same species with substantially different susceptibility profiles were considered distinct strains.

### Statistical analysis

Risk factor analyses were conducted using SPSS 29.0 (IBM Corp., Armonk, New York). The chi-squared test and binary logistic regression were used for nominal variables and the Mann–Whitney U test for scale variables in the univariate analysis. For MDRO carriage, two separate analyses were conducted: one including all patients (Table 1), and the other excluding those residing in the country of hospitalization (Supplementary Table S1). We also performed separate MDRO-type specific multivariable analyses for ESBL-PE and MRSA (Supplementary Tables S2 and S3). Variables with *P* < 0.2 were included for the multivariate logistic regression; for highly correlated pairs, only one variable was retained. Akaike information criteria were applied to find the most parsimonious model.

**Table 1:** Demographics and risk factor analysis of colonization by multidrug-resistant organisms (MDRO) among children hospitalized abroad and screened in Helsinki University Hospital, January 2010—December 2024

	Patients n = 459 (%^a^)	MDRO + n = 158 (%^a^)	MDRO − n = 301 (%^a^)	OR (95% CI) in univariate analysis	p value in univariate analysis	AOR (95% CI) in multivariable analysis^b^	p value in multivariable analysis
**Sex**							
Male	267 (58.2)	86 (32.2)	181 (67.8)	Ref.	Ref.	NI	NI
Female	192 (41.8)	72 (37.5)	120 (62.5)	1.3 (0.9–1.9)	0.24	NI	NI
**Age groups (years)**					0.54		
<1 y	119 (25.9)	40 (33.6)	79 (66.4)	Ref.	Ref.	NI	NI
1 to <6y	170 (37.0)	65 (38.2)	105 (61.8)	1.2 (0.7–2.0)	0.42	NI	NI
6 to <12y	82 (17.9)	24 (29.3)	58 (70.7)	0.8 (0.4–1.5)	0.52	NI	NI
12 to <18y	88 (19.2)	29 (33.0)	59 (67.0)	1.0 (0.5–1.7)	0.92	NI	NI
**CCI**					0.04	Eliminated^c^	Eliminated^c^
0 points	353 (76.9)	123 (34.8)	230 (65.2)	Ref.	Ref.	NI	NI
1 point	49 (10.7)	9 (18.4)	40 (81.6)	0.4 (0.2–0.9)	0.03	NI	NI
2 points	45 (9.8)	20 (44.4)	25 (55.6)	1.5 (0.8–2.8)	0.21	NI	NI
3 or more points	12 (2.6)	6 (50.0)	6 (50.0)	1.9 (0.6–5.9)	0.29	NI	NI
**Travel type**					<0.001		<0.001
Leisure/other	146 (31.8)	21 (14.4)	125 (85.6)	Ref.	Ref.	Ref.	Ref.
Residence abroad	188 (41.0)	70 (37.2)	118 (62.8)	3.5 (2.0–6.1)	<0.001	3.1 (1.7–5.8)	<0.001
Visiting friends and relatives	125 (27.2)	67 (53.6)	58 (46.4)	6.9 (3.8–12.3)	<0.001	2.9 (1.5–5.7)	0.002
**Geographic region**					<0.001		
North America	8 (1.7)	0 (0)	8 (100)	NA	NA	NI	NI
Latin America and Caribbean	7 (1.5)	3 (42.9)	4 (57.1)	3.4 (0.7–15.5)	0.12	NI	NI
Sub-Saharan Africa	41 (8.9)	32 (78.0)	9 (22.0)	15.9 (7.2–35.5)	<0.001	NI	NI
North Africa and Middle East	60 (13.1)	33 (55.0)	27 (45.0)	5.5 (3.0–9.9)	<0.001	NI	NI
Asia	68 (14.8)	40 (58.8)	28 (41.2)	6.4 (3.6–11.3)	<0.001	NI	NI
Oceania	1 (0.2)	0 (0)	1 (100)	NA	NA	NI	NI
Europe	274 (59.7)	50 (18.2)	224 (81.8)	Ref.	Ref.	NI	NI
**Country income level**					<0.001		<0.001
High income	245 (53.4)	33 (13.5)	212 (86.5)	Ref.	Ref.	Ref.	Ref.
Upper middle income	118 (25.7)	55 (46.6)	63 (53.4)	5.6 (3.4–9.4)	<0.001	5.2 (3.0–9.1)	<0.001
Lower middle income	72 (15.7)	49 (68.1)	23 (31.9)	13.7 (7.4–25.4)	<0.001	11.8 (6.1–22.8)	<0.001
Low income	24 (5.2)	21 (87.5)	3 (12.5)	45.0 (12.7–159.2)	<0.001	38.8 (10.5–143.1)	<0.001
**Direct interhospital transfer**						Eliminated^c^	Eliminated^c^
Yes	102 (22.2)	21 (20.6)	81 (79.4)	0.4 (0.2–0.7)	0.001	NI	NI
No	357 (77.8)	137 (38.4)	220 (61.6)	Ref.	Ref.	NI	NI
**ICU treatment abroad**							
Yes	83 (18.1)	24 (28.9)	59 (71.1)	0.7 (0.4–1.2)	0.24	NI	NI
No	376 (81.9)	134 (35.6)	242 (64.4)	Ref.	Ref.	NI	NI
**Major invasive procedure abroad**						Eliminated^c^	Eliminated^c^
Yes	150 (32.7)	43 (28.7)	107 (71.3)	0.7 (0.4–1.0)	0.07	NI	NI
No	309 (67.3)	115 (37.2)	194 (62.8)	Ref.	Ref.	NI	NI
**Antibiotic use abroad**							
Yes	196 (42.7)	83 (42.3)	113 (57.7)	1.8 (1.2–2.7)	0.002	2.2 (1.4–3.6)	<0.001
No/not recorded	263 (57.3)	75 (28.5)	118 (71.5)	Ref.	Ref.	Ref.	Ref.
**Antibiotic use during screening**							
Yes	58 (12.6)	22 (37.9)	36 (62.1)	1.2 (0.7–2.1)	0.55	NI	NI
No	401 (87.4)	136 (33.9)	265 (66.1)	Ref.	Ref.	NI	NI
**Length of hospital stay** ^d^						Eliminated^c^	Eliminated^c^
Median days (IQR)	5 (3.0–12.0)	7.0 (3.0–14.0)	4.0 (2.0–9.0)	1.02 (1.01–1.04)	0.01	NI	NI

^a^Percentages in the ‘Patients’ column represent column percentages (proportion of the total study population). Percentages in the ‘MDRO+’ and ‘MDRO–’ columns represent row percentages (proportion within each variable category).

^b^The following variables were included in the multivariable analysis: CCI, travel type, country income level, length of hospital stay, direct interhospital transfer, major invasive procedure abroad and antibiotic use abroad. Country income level was chosen to multivariate analysis over geographical region due to the stronger correlation in univariate analysis.

^c^Eliminated before the final step in backward selection.

^d^Length of hospital stay data was available for 267 patients, of whom 82 (30.7%) were MDRO colonized.

Abbreviations: AOR, adjusted odds ratio; CI, confidence interval; ICU, intensive care unit; NA, not applicable; NI, not included; OR, odds ratio; Ref, reference.

### Ethical statement

According to the Finnish Medical Research Act, register-based studies do not need a review by an ethics board. The protocol was approved by the research board of the HUS Department of Internal Medicine.

## Results

### Study population

Between 1 January 2010 and 31 December 2024, 11 799 patients were screened for both MRGN and MRSA. After exclusions (no history of hospitalization abroad, age > 18 years, incomplete screening and/or hospitalization/travel in multiple areas), 459 patients were included in the analyses.

The characteristics of the study population are summarized in Table 1. Nearly half of all hospitalizations (47%) occurred in LMICs, although most children (60%) had been hospitalized in Europe. The majority of patients either lived abroad (41%) or were visiting friends or relatives (27%). The median age was 3 years, 62% were younger than 6 years, and most had no comorbidities (77% with a CCI of 0). The median hospital stay abroad was 5 days. Antibiotic use was documented in 43%, while 18% had required ICU care, and 33% had undergone invasive procedures. In 72.1% of patients, the reasons for hospitalization abroad and at HUS were the same, and only 22% arrived through direct interhospital transfer.

### Multidrug-resistant bacteria

Colonization with at least one MDRO was detected in 34.4% (158/459) of patients, with the annual MDRO rates varying between 14.3% and 52.9%; the increase over the study period proved statistically significant (Supplementary Table S4). ESBL-PE colonization was identified in 133 of the 158 MDRO-colonized patients (84.2%), with a total of 187 ESBL-PE isolates detected. *Escherichia coli* (n = 140, 74.9%) and *K. pneumoniae* (n = 33, 17.6%) were the most common ESBL-PE species. Co-carriage with several ESBL-PE strains was observed in 41/133 (30.8%) patients, 29 of whom carried two different species and 12 carried bacteria of the same species with two different AMR phenotypes.

**Figure 1:**
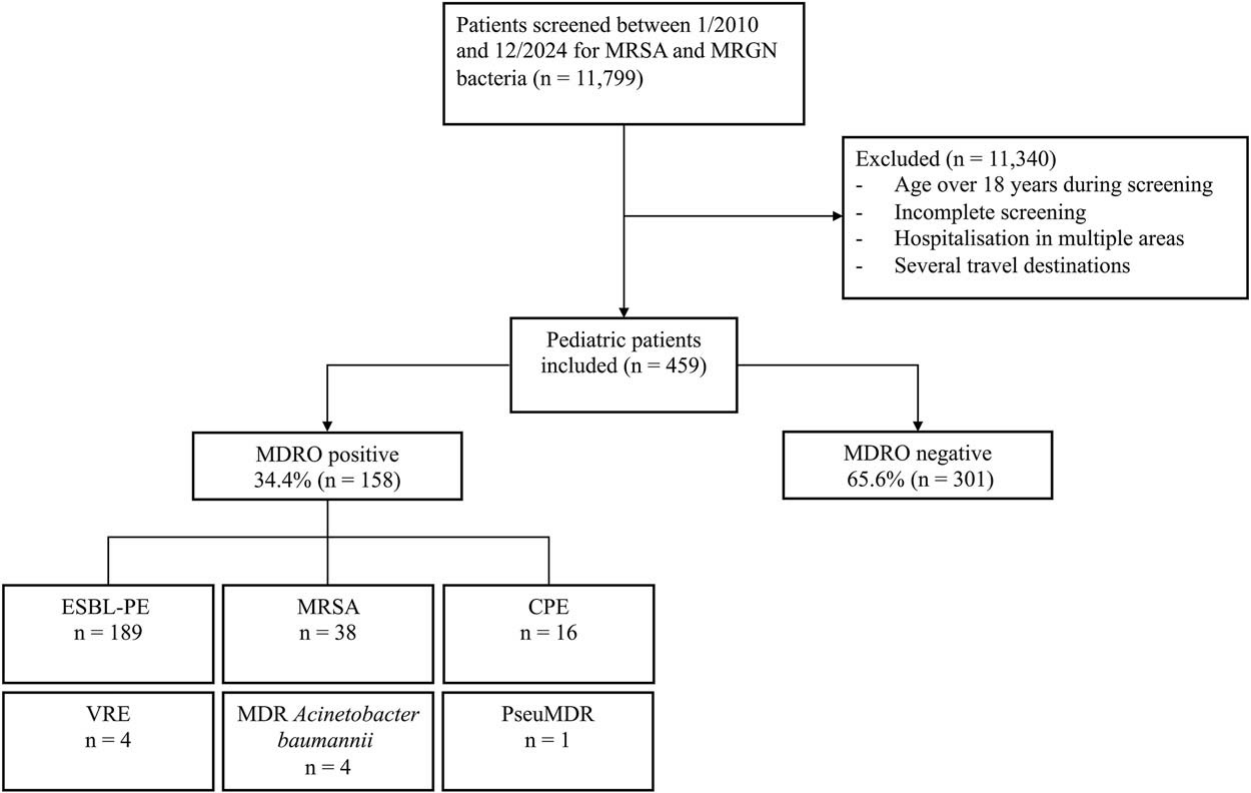
Flowchart showing multidrug-resistant bacteria detected in samples from Finnish children hospitalized abroad and screened in 2010–2024 at Helsinki University Hospital, Finland. n = number of patients. A single patient may have more than one strain of the same MDR bacterial class. MDR bacterial classes: ESBL-PE, MRSA, CPE, VRE, MDR *Acinetobacter baumannii* and MDR *Pseudomonas aeruginosa*

MRSA was identified in 35 (7.6%), MDRACI in three (0.7%) and MDRPA in one (0.2%) of the 459 patients (Figure 1). CPE colonization was recorded in 14 (3.1%) patients with a total of 16 isolates; 10 *E. coli*, three *K. pneumoniae* and three other species. The patients had been hospitalized mostly (13/14) in LMICs; four in Turkey, three in Somalia, two in India and one each in Colombia, Egypt, Pakistan, Romania and Ukraine. New Delhi metallo-beta-lactamase (NDM) genes were detected in nine isolates (including five *E. coli* and four other species); OXA-type carbapenemase genes in six (five *E. coli*, one *K. pneumoniae*), and KPC gene was detected in one *K. pneumoniae* isolate. At least nine of the 14 CPE-positive patients had received antibiotics abroad.

A total of 250 of the 459 patients were screened for VRE, four of whom (1.6%) were found to be carriers (all *Enterococcus faecium* with VanA gene) (Figure 1).

### Risk factors for colonization

Several factors were significantly associated with MDRO colonization in univariate analysis: travel type, hospitalization region, hospitalization country income level, Charlson comorbidity index, antibiotic use and length of hospital stay abroad. Of the three travel types, VFR travel and foreign residence were associated with higher odds of colonization than leisure or other travel. The MDRO colonization rates decreased by income level from 87.5% (21/24) in low-income countries to 68.1% (49/72) in lower-middle, 46.6% (55/118) in upper-middle and 13.5% (33/245) in high-income countries. An inverse relationship was observed between direct interhospital transfer and MDRO colonization (Table 1). Country- and region-specific proportions of MDRO colonization are shown in Supplementary table S5.

In multivariable analysis hospitalization country’s income level (*P* < 0.001), travel type (*P* < 0.001) and antibiotic use abroad (*P* = 0.002) were identified as independent risk factors for MDRO colonization. A strong correlation with MDRO colonization risk was seen with hospitalization country’s decreasing income level both globally and within geographical regions (Table 2).

**Table 2:** Impact of country income level: MDR bacterial colonization status of children hospitalized abroad stratified by income level of the hospitalization country, January 2010—December 2024

	Patients n (%^a^)	MDRO + n (%^a^)	MDRO − n (%^a^)	OR (95% CI) in univariate analysis	p value in univariate analysis
**Country income level—All regions (n = 458)**					<.0.001
High income	245 (53.4)	33 (13.5)	212 (86.5)	Ref.	Ref.
Upper middle income	118 (25.7)	55 (46.6)	63 (53.4)	5.6 (3.4–9.4)	<0.001
Lower middle income	72 (15.7)	49 (68.1)	23 (31.9)	13.7 (7.4–25.4)	<0.001
Low income	24 (5.2)	21 (87.5)	3 (12.5)	45.0 (12.7–159.2)	<0.001
**Country income level—Europe (n = 274)**					<0.001
High income	229 (83.6)	31 (13.5)	198 (86.5)	Ref.	Ref.
Upper middle income	35 (12.8)	14 (40.0)	21 (60.0)	4.3 (2.0–9.2)	<0.001
Lower middle income	10 (3.6)	5 (50.0)	5 (50.0)	6.4 (1.7–23.3)	0.005
**Country income level—Sub-Saharan Africa (n = 41)**					0.15
Upper middle income	3 (7.3)	1 (33.3)	2 (66.7)	Ref.	Ref.
Lower middle income	15 (36.6)	11 (73.3)	4 (26.7)	5.5 (0.4–78.6)	0.06
Low income	23 (56.1)	20 (87.0)	3 (13.0)	13.3 (0.9–196.4)	0.21
**Country income level—North Africa and Middle East (n = 60)**					
High income	4 (6.7)	1 (25.0)	3 (75.0)	NA	NA
Upper middle income	41 (68.3)	23 (56.1)	18 (43.9)	Ref.	Ref.
Lower middle income	15 (25.0)	9 (60.0)	6 (40.0)	1.2 (0.4–3.9)	0.79
**Country income level—Asia (n = 68)**					
High income	3 (4.4)	1 (33.3)	2 (66.7)	NA	NA
Upper middle income	32 (47.1)	14 (43.8)	18 (56.3)	Ref.	Ref.
Lower middle income	32 (47.1)	23 (71.9)	9 (28.1)	3.3 (1.2–9.3)	0.03
Low income	1 (1.4)	0 (0)	1 (100)	NA	NA

^a^Percentages in the ‘Patients’ column represent column percentages (proportion of the total study population). Percentages in the ‘MDRO+’ and ‘MDRO–’ columns represent row percentages (proportion within each variable category).

Abbreviations: CI, confidence interval; NA, not applicable; OR, odds ratio; Ref, reference.

We conducted a separate multivariate risk factor analysis among visitors (VFR or leisure travellers), thus excluding the residents of the hospitalization country. The results mirrored those of the whole study population with the exception that age of under one year emerged as an additional statistically significant risk factor in this subset (*P* = 0.03) (Supplementary Table S1).

In additional MDRO type-specific multivariable analyses, risk factors for ESBL-PE colonization were the same as for MDRO overall reflecting the high proportion of ESBL-PE-colonized patients in the study population (133/158) (Supplementary Table S2). For MRSA, the only statistically significant risk factors identified were increasing CCI and decreasing country income level (Supplementary Table S3).

### MDR infections

Among the 158 colonized patients, five (3.2%) presented with a clinical MDRO infection during hospitalization in Finland: two with urinary tract infections (UTI), two with surgical site wound infections and one with intra-abdominal infection.

## Discussion

During the over 15-year surveillance period, we screened 459 children hospitalized abroad and found that one-third carried MDROs. Colonization was significantly associated with hospitalization country income level, antibiotic use abroad, VFR travel and residence in the hospitalization country. To our knowledge, this is the first study to identify risk factors for MDRO colonization among paediatric patients hospitalized abroad.

### Colonization by intestinal MDR bacteria

The carriage rate of 34.4% in the present study resembles the MDR rates in our previous studies—conducted among mostly adult patients—exploring Finnish travellers after hospitalization abroad.^11–13^ In our paediatric cohort, ESBL-PE was the most prevalent MDR type, with a colonization rate of 29.0%. This is substantially higher than the background rates reported in Finland: Previous studies among adults found ESBL-PE colonization rates of 1.2% and 4.5% in pre-travel samples of Finnish travellers collected 2009–2010 and 2015–2017, respectively.^7^^,^^26^ This supports the assumption that the vast majority of MDROs reported in our study were acquired abroad. The high rates in our study align with our findings in hospitalized travellers.^11–13^

MRSA was detected in 7.6% of children in our cohort, which is a high rate considering that in Finland, even in clinical samples, only 3% of *S. aureus* strains carry the *mecA* gene.^27^ VRE (all *E. faecium*) was found only in 1.6% of patients screened for it. While no data exists on community VRE colonization rates in Finland, a Swedish study found 0% carriage among healthy children.^28^ Given that only 13 cases were reported from clinical samples across Finland in 2023, the background prevalence among community dwellers is likely negligible, similar to Sweden.^27^ The CPE colonization rate of 3.1% was concerning, given the low CPE incidence in our hospital district.^29^

### Risk factors

We found a strong inverse association between national income level of the country of hospitalization and subsequent MDRO colonization. Previous studies on patients hospitalized abroad have mainly linked MDRO colonization risk to geographical regions, with South and South-East Asia and Africa, consistently identified as the highest-risk areas.^11–13^ To our knowledge, this is the first study to show decreasing national income level itself as an independent risk factor. This aligns with global surveillance data indicating that AMR burden is greatest in poorer regions,^2^ where limited resources combined with weak regulation, inadequate infection control, high antibiotic use and poor sanitation foster AMR spread.^30^

Our 75% ESBL-PE colonization rate among children hospitalized in low-income countries is comparable to rates reported in local paediatric patients in Madagascar (57%),^31^ Niger (61%),^32^ Ethiopia (61%)^33^ and Rwanda (93%).^34^

Antibiotic use during hospitalization abroad was a significant risk factor for MDR bacterial colonization, consistent with numerous paediatric^20,35–37^ and adult studies.^6,7,9^

Unlike in our mixed cohorts, this study was dominated by foreign residents (41%), largely due to referrals for paediatric care from other European countries such as Estonia. Estonia was particularly overrepresented because of a bilateral agreement allowing Estonian paediatric patients to receive advanced medical care in Finland. Owing to this overrepresentation, the colonization rate reported for Europe may appear comparatively low: only 8.0% of Estonian patients were MDRO colonized compared with the European average of 18.4%. Foreign residents had longer hospital stays (mean 19 days vs 7 days for both VFR and leisure travellers) and higher CCI (mean 0.6 vs 0.3 for both VFR and leisure travellers), reflecting their more complex medical conditions.

To omit the influence of the large subgroup with foreign residency, we conducted a separate analysis excluding residents of the hospitalization country. This analysis revealed age under 1 year as an additional risk factor, while all the other associations remained unchanged. This finding concurs with prior reports linking young age to higher MDRO colonization rates,^33,38–41^ and aligns with our previous observation that age under six years is a risk factor for MDRO colonization after hospitalization abroad.^11^

VFR travellers comprised 27.0% of our cohort, the proportion markedly higher than in our previous mixed-age cohorts: 11.2% in 2010–2013^11^ and 7.0% in 2010–2019.^12^ Such VFR overrepresentation is expected as children travel less for other reasons, whereas VFR travel is by definition family- and relationship-driven mobility and often includes younger family members as well.^10^ VFR travel emerged as an independent risk factor for MDR colonization, a finding previously shown among adults,^6,11,42,43^ but not among children. Both VFR travellers and residents have longer and presumably more extensive exposure to local MDROs.

A short time since hospitalization abroad^13^ and direct interhospital transfer have previously been identified as risk factors for MDRO colonization,^11^ but in our data the association appeared inverse. This is most likely explained by the fact that a substantially larger proportion (76%) of the directly transferred patients had been hospitalized in high-income countries.

### Clinical infections

In our study, 3.2% of MDR-colonized children developed clinical MDR infections, a rate substantially lower than reported in Finnish mixed-age cohorts (11.4%^11^ and 10.6%^12^), but comparable to the 3.6% observed among paediatric refugees in a German hospital,^44^ where 33.8% (110/325) were MDR colonized.^44^ Despite the low rate, the burden of clinical MDR infections among infants are well documented.^1,2^ The lower rate in our cohort may reflect the limited inclusion of neonates, since birth and early travel abroad are rare. Families may also delay travel after severe illness.

### Limitations

As a retrospective study, the data was restricted to those recorded in the patient files. Descriptions on hospitalization abroad had limited details and, therefore, ICU stays, antibiotic use and other travel-related information may be under-recorded. Pre-travel MDR carriage or post-travel acquisition cannot be excluded but both are unlikely given Finland’s low prevalence: for ESBL-PE under 5%^7,26,45^ and for MRSA 3% of clinical *S. aureus* isolates^27^ (suggesting community rates < 1%).

As screening occurred up to 12 months post-discharge, some patients may have cleared colonization before testing: ESBL-PE colonization is often transient^46^ and carriage rates decrease substantially within months,^6,8^ however, the majority (77%) were screened within the first three months.

Only microbiologically confirmed infections were included, potentially underestimating the actual MDRO infection rates.

Finally, the lack of a non-hospitalized control group limits the ability to differentiate nosocomial from community-acquired MDROs abroad.

## Conclusion

Our study demonstrates that MDRO colonization in paediatric patients after hospitalization abroad is both frequent and strongly influenced by the income level of the hospitalization country. VFR travel, residence abroad and antibiotic use abroad represent important additional risk factors. These results highlight the need for enhanced screening and targeted infection control measures for children returning from care abroad, particularly those hospitalized in LMICs or with high-risk travel exposures.

## Supplementary Material

taag003_Revised_supplementary_tables
