# Supplementary material for: Colonization with multidrug-resistant bacteria among children hospitalized abroad—a study from Finland
Source: J Travel Med. 2026 Jan 22;33(3):taag003. doi: 10.1093/jtm/taag003 (PMC13042226; doi:10.1093/jtm/taag003)
Supplement: taag003_Revised_supplementary_tables [file taag003_revised_supplementary_tables.pdf]

Supplementary table 1. Demographics and risk factor analysis of colonization by multidrug-resistant organisms (MDRO) among children hospitalized abroad during leisure or VFR (visiting friends and relatives) travel (patients with foreign residence at time of hospitalization excluded), January 2010 – December 2024.

	Patients n=271 (%[a])	MDRO + n=87 (%[a])	MDRO - n=184 (%[a])	OR (95% CI) in univariate analysis	p value in univariate analysis	AOR (95% CI) in multivariable analysis[c]	p value in multivariable analysis
**Sex**							
Male	159 (58.7)	47 (29.6)	112 (70.4)	Ref.	Ref.	NI	NI
Female	112 (41.3)	40 (35.7)	72 (64.3)	1.3 (0.8-2.2)	0.29	NI	NI
**Age groups (years)**					0.08	**Eliminated**[b]	**Eliminated**[b]
<1 y	37 (13.7)	18 (48.6)	19 (51.4)	Ref.	Ref.	NI	NI
1 to <18y	107 (39.5)	36 (33.6)	71 (66.4)	0.5 (0.2-1.1)	0.11	NI	NI
6 to <12y	63 (23.2)	16 (25.4)	47 (74.6)	0.4 (0.2-0.8)	0.02	NI	NI
12 to <18y	64 (23.6)	17 (26.6)	47 (73.4)	0.4 (0.2-0.9)	0.03	NI	NI
**Age**							
Infant (<1 year old)	37 (13.7)	18 (48.6)	19 (51.4)	2.2 (1.1-4.5)	0.03	2.4 (1.0-5.9)	0.05
Other (1 to <18y)	234 (86.3)	69 (29.5)	165 (70.5)	Ref.	Ref.	Ref.	Ref.
**CCI**					0.88		
0 points	231 (85.2)	73 (31.6)	158 (68.4)	Ref.	Ref.	NI	NI
1 point	16 (5.9)	5 (31.3)	11 (68.8)	1.0 (0.3-2.9)	NA	NI	NI
2 points	17 (6.3)	7 (41.2)	10 (58.8)	1.5 (0.6-4.1)	0.42	NI	NI
3 points	7 (2.6)	2 (28.6)	5 (71.4)	0.9 (0.2-4.6)	0.87	NI	NI
**Travel type**							
Leisure/other	146 (53.9)	21 (14.4)	125 (85.6)	Ref.	Ref.	Ref.	Ref.
Visiting friends and relatives	125 (46.1)	66 (52.8)	59 (47.2)	6.7 (3.7-11.9)	<0.001	2.8 (1.3-5.7)	0.006
**Geographic region**					<0.001		
North America	5 (1.8)	0 (0)	5 (100)	NA	NA	NI	NI

Latin America and Caribbean	5 (1.8)	2 (40.0)	3 (60.0)	4.1 (0.6-25.8)	0.14	NI	NI
Sub-Saharan Africa	23 (8.5)	20 (87.0)	3 (13.0)	40.6 (11.1-148.8)	<0.001	NI	NI
North Africa and Middle East	44 (16.2)	22 (50.0)	22 (50.0)	6.1 (2.9-12.9)	<0.001	NI	NI
Asia	45 (16.6)	22 (48.9)	23 (51.1)	5.8 (2.8-12.3)	<0.001	NI	NI
Europe	149 (55.0)	21 (14.1)	128 (85.9)	Ref.	Ref.	NI	NI
**Country income level**					<0.001		<0.001
High income	133 (49.1)	15 (11.3)	118 (88.7)	Ref.	Ref.	Ref.	Ref.
Upper middle income	81 (29.9)	30 (37.0)	51 (63.0)	4.6 (2.3-9.3)	<0.001	3.3 (1.5-7.1)	0.003
Lower middle income	40 (14.8)	26 (65.0)	14 (35.0)	14.6 (6.3-33.9)	<0.001	12.4 (4.6-33.5)	<0.001
Low income	17 (6.3)	16 (94.1)	1 (5.9)	125.9 (15.6-1018.1)	<0.001	88.9 (10.2-776.3)	<0.001
**Direct interhospital transfer**						**Eliminated**[b]	**Eliminated**[b]
Yes	59 (21.8)	11 (18.6)	48 (81.4)	0.4 (0.2-0.8)	0.01	NI	NI
No	212 (78.2)	76 (35.8)	136 (64.2)	Ref.	Ref.	NI	NI
**ICU treatment abroad**							
Yes	28 (10.3)	8 (28.6)	20 (71.4)	0.8 (0.4-2.0)	0.67	NI	NI
No	243 (89.7)	79 (32.5)	164 (67.5)	Ref.	Ref.	NI	NI
**Major invasive procedure abroad**						**Eliminated**[b]	**Eliminated**[b]
Yes	79 (29.2)	16 (20.3)	63 (79.7)	0.4 (0.2-0.8)	0.008	NI	NI
No	192 (70.8)	71 (37.0)	121 (63.0)	Ref.	Ref.	NI	NI
**Antibiotic use abroad**							
Yes	117 (43.2)	50 (42.7)	67 (57.3)	2.4 (1.4-4.0)	0.001	3.0 (1.5-5.8)	0.001
No/not recorded	154 (56.8)	37 (24.0)	117 (76.0)	Ref.	Ref.	Ref.	Ref.

**Antibiotic use during screening**							
Yes	34 (12.5)	10 (29.4)	24 (70.6)	0.9 (0.4-1.9)	0.72	NI	NI
No	237 (87.5)	77 (32.5)	160 (67.5)	Ref.	Ref.	NI	NI
**Length of hospital stay[d]**						**Eliminated[b]**	**Eliminated[b]**
Median days (IQR)	4 (2.0-7.0)	5 (3.0-12.5)	4 (2.0-6.3)	1.03 (1.00-1.06)	0.05	NI	NI

[a] Percentages in the “Patients” column represent column percentages (proportion of the total study population). Percentages in the “MDRO+” and “MDRO–” columns represent row percentages (proportion within each variable category).

[b] Eliminated before the final step in backward selection.

[c] The following variables were included in the multivariable analysis: age, travel type, country income level, length of hospital stay, direct interhospital transfer, major invasive procedure abroad and antibiotic use abroad. Country income level was chosen to multivariate analysis over geographical region due to the stronger correlation in univariate analysis.

[d] Length of hospital stay data was available for 194 patients, of whom 60 (30.9%) were MDRO colonised.

Abbreviations: AOR, adjusted odds ratio; CI, confidence interval; ICU, intensive care unit; NA, not applicable; NI, not included; OR, odds ratio; Ref, reference.

Alt text: Supplementary Table 1 shows demographic characteristics and the results of univariate and multivariate risk factor analyses for 271 patients travelling to visit friends and relatives or for leisure of whom 87 (32.1%) were MDRO positive. Risk factors for MDRO colonization in this subgroup were age under 1 year, visiting friends and relatives, decreasing country income level and antibiotic use abroad.

Supplementary table 2 Demographics and risk factor analysis of ESBL-producing Enterobacterales (ESBL-PE) colonization among children hospitalized abroad, January 2010 – December 2024

	ESBL + n=133 (%)	ESBL – n=326 (%)	OR (95% CI) in univariate analysis	p value in univariate analysis	AOR (95% CI) in multivariable analysis[a]	p value in multivariable analysis
**Sex**						
Male	73 (27.3)	194 (72.7)	Ref.	Ref.	NI	NI
Female	60 (31.3)	132 (68.8)	1.2 (0.8-1.8)	0.36	NI	NI
**Age groups (years)**				0.34		
<1 y	29 (24.4)	90 (75.6)	Ref.	Ref.	NI	NI
1 to <6y	57 (33.5)	113 (66.5)	1.6 (0.9-2.6)	0.10	NI	NI
6 to <12y	21 (25.6)	61 (74.4)	1.1 (0.6-2.0)	0.84	NI	NI
12 to <18y	26 (29.5)	62 (70.5)	1.3 (0.7-2.4)	0.41	NI	NI
**CCI**				0.06	**Eliminated[b]**	**Eliminated[b]**
0 points	104 (29.5)	249 (70.5)	Ref.	Ref.	NI	NI
1 point	7 (14.3)	42 (85.7)	0.4 (0.2-0.9)	0.03	NI	NI
2 points	17 (37.8)	28 (62.2)	1.5 (0.8-2.8)	0.26	NI	NI
3 points	5 (41.7)	7 (58.3)	1.7 (0.5-5.5)	0.37	NI	NI
**Travel type**				<0.001		0.02
Leisure/other	19 (13.0)	127 (87.0)	Ref.	Ref.	Ref.	Ref.
Residence abroad	57 (30.3)	131 (69.7)	2.9 (1.6-5.2)	<0.001	2.4 (1.3-4.5)	0.007
Visiting friends and relatives	57 (45.6)	68 (54.4)	5.6 (3.1-10.2)	<0.001	2.3 (1.2-4.5)	0.02
**Geographic region**				<0.001	**Eliminated[b]**	**Eliminated[b]**
North America	0 (0)	8 (100)	NA	NA	NI	NI
Latin America and the Caribbean	3 (42.9)	4 (57.1)	4.5 (1.0-21.0)	0.05	NI	NI
Sub-Saharan Africa	30 (73.2)	11 (26.8)	16.4 (7.6-35.5)	<0.001	NI	NI
North Africa and Middle East	26 (43.3)	34 (56.7)	4.6 (2.5-8.5)	<0.001	NI	NI
Asia	35 (51.5)	33 (48.5)	6.4 (3.6-11.5)	<0.001	NI	NI
Oceania	0 (0)	1 (100)	NA	NA	NI	NI
Europe	39 (14.2)	235 (85.8)	Ref.	Ref.	NI	NI
**Country income level**				<0.001		<0.001
High income	25 (10.2)	220 (89.8)	Ref.	Ref.	Ref.	Ref.
Upper middle income	47 (39.8)	71 (60.2)	5.8 (3.3-10.1)	<0.001	5.4 (3.0-9.7)	<0.001
Lower middle income	43 (59.7)	29 (40.3)	13.0 (7.0-24.4)	<0.001	11.6 (5.9-22.7)	<0.001
Low income	18 (75.0)	6 (25.0)	26.4 (9.6-72.7)	<0.001	23.1 (8.0-66.5)	0.001
**Direct interhospital transfer**					**Eliminated[b]**	**Eliminated[b]**
Yes	19 (18.6)	83 (81.4)	0.5 (0.3-0.8)	0.01	NI	NI

No	114 (31.9)	243 (68.1)	Ref.	Ref.	NI	NI
**ICU treatment abroad**						
Yes	20 (24.1)	63 (75.9)	0.7 (0.4-1.3)	0.28	NI	NI
No	113 (30.1)	263 (69.9)	Ref.	Ref.	NI	NI
**Major invasive procedure abroad**					**Eliminated[b]**	**Eliminated[b]**
Yes	33 (22.0)	117 (78.0)	0.6 (0.4-0.9)	0.02	NI	NI
No	100 (32.4)	209 (67.6)	Ref.	Ref.	NI	NI
**Antibiotic use abroad**						
Yes	72 (36.7)	124 (63.3)	1.9 (1.3-2.9)	0.002	2.3 (1.4-3.6)	<0.001
No/not recorded	61 (23.2)	202 (76.8)	Ref.	Ref.	Ref.	Ref.
**Antibiotic use during screening**					**Eliminated[b]**	**Eliminated[b]**
Yes	21 (36.2)	37 (63.8)	1.5 (0.8-2.2)	0.20	NI	NI
No	112 (27.9)	289 (72.1)	Ref.	Ref.	NI	NI
**Length of hospital stay[c]**						
Median days (IQR)	7 (3.0-14.0)	4 (2.0-11.0)	1.01 (0.99-1.03)	0.25	NI	NI

[a] The following variables were included in the multivariable analysis: CCI, travel type, geographical region, country income level, length of hospital stay, direct interhospital transfer, major invasive procedure abroad, antibiotic use abroad and antibiotic use during screening.
[b] Eliminated before the final step in backward selection.
[c] Length of hospital stay data was available for 267 patients, of whom 82 (30.7%) were MDRO-colonised.

Abbreviations: AOR, adjusted odds ratio; CI, confidence interval; ICU, intensive care unit; NA, not applicable; NI, not included; OR, odds ratio; Ref, reference.

Alt text: Supplementary table 2 is summarizing the study population's demographic characteristics and the results of univariate and multivariate risk factor analyses for ESBL colonization. Risk factors for ESBL colonization; VFR-travel, residence abroad, decreasing country income level and antibiotic use abroad.

Supplementary table 3 Demographics and risk factor analysis of methicillin-resistant Staphylococcus aureus (MRSA) colonization status among children hospitalized abroad, January 2010 – December 2024

	MRSA + n=35 (%)	MRSA – n=423 (%)	OR (95% CI) in univariate analysis	p value in univariate analysis	AOR (95% CI) in multivariable analysis[a]	p value in multivariable analysis
**Sex**						
Male	17 (6.4)	250 (93.6)	Ref.	Ref.	NI	NI
Female	18 (9.4)	173 (90.6)	1.5 (0.8-3.1)	0.23	NI	NI
**Age groups (years)**				0.94		
<1 y	8 (6.7)	111 (93.3)	Ref.	Ref.	NI	NI
1 to <6y	13 (7.7)	156 (92.3)	1.2 (0.5-2.9)	0.76	NI	NI
6 to <12y	6 (7.3)	76 (92.7)	1.1 (0.4-3.3)	0.87	NI	NI
12 to <18y	8 (9.1)	80 (90.9)	1.4 (0.5-3.9)	0.53	NI	NI
**CCI**				0.02		0.003
0 points	23 (6.5)	329 (93.5)	Ref.	Ref.	Ref.	Ref.
1 point	3 (6.1)	46 (93.9)	0.9 (0.3-3.2)	0.91	1.8 (0.5-6.6)	0.40
2 points	5 (11.1)	40 (88.9)	1.8 (0.6-5.0)	0.27	2.6 (0.9-7.9)	0.08
3 points	4 (33.3)	8 (66.7)	7.2 (2.0-25.5)	0.002	13.4 (3.1-57.2)	<0.001
**Travel type**						
Leisure/other	0 (0)	146 (100)	NA	NA	NI	NI
Residence abroad	20 (10.6)	168 (89.4)	0.9 (0.4-1.8)	0.69	NI	NI
Visiting friends and relatives	15 (12.1)	109 (87.9)	Ref.	Ref.	NI	NI
**Geographical region**				0.007	Eliminated[b]	Eliminated[b]
North America	0 (0)	8 (100)	NA	NA	NI	NI
Latin America and the Caribbean	0 (0)	7 (100)	NA	NA	NI	NI
Sub-Saharan Africa	9 (22.0)	32 (78.0)	6.7 (2.6-17.4)	<0.001	NI	NI
North Africa, Middle East	9 (15.0)	51 (85.0)	4.2 (1.7-10.7)	0.002	NI	NI
Asia	6 (8.8)	62 (91.2)	2.3 (0.8-6.5)	0.11	NI	NI
Oceania	0 (0)	1 (100)	NA	NA	NI	NI
Europe	11 (4.0)	262 (96.0)	Ref.	Ref.	NI	NI
**Country income level**				<0.001		<0.001
High income	7 (2.9)	238 (97.1)	Ref.	Ref.	Ref.	Ref.
Upper middle income	12 (10.3)	105 (89.7)	3.9 (1.5-10.1)	<0.001	3.6 (1.3-9.8)	0.01
Lower middle income	9 (12.5)	63 (87.5)	4.9 (1.7-13.6)	0.003	4.4 (1.5-13.1)	0.007
Low income	7 (29.2)	17 (70.8)	14.0 (4.4-44.5)	0.006	19.9 (5.7-69.0)	<0.001
**Direct interhospital transfer**						

Yes	2 (2.0)	100 (98.0)	0.2 (0-0.8)	0.03	0.3 (0.1-1.2)	0.10
No	33 (9.3)	323 (90.7)	Ref.	Ref.	Ref.	Ref.
**ICU treatment abroad**					**Eliminated[b]**	**Eliminated[b]**
Yes	2 (2.4)	81 (97.6)	0.3 (0.1-1.1)	0.07	NI	NI
No	33 (8.8)	342 (91.2)	Ref.	Ref.	NI	NI
**Major invasive procedure abroad**						
Yes	11 (7.3)	139 (92.7)	0.9 (0.4-2.0)	0.94	NI	NI
No	24 (7.8)	284 (92.9)	Ref.	Ref.	NI	NI
**Antibiotic use abroad**						
Yes	18 (9.2)	178 (90.8)	1.5 (0.7-2.9)	0.29	NI	NI
No/not recorded	17 (6.5)	245 (93.5)	Ref.	Ref.	NI	NI
**Antibiotic use during screening**						
Yes	1 (1.7)	57 (98.3)	0.2 (0-1.4)	0.10	0.2 (0.0-1.8)	0.15
No	34 (8.5)	367 (91.5)	Ref.	Ref.	Ref.	Ref.
**Length of hospital stay[c]**						
Median days (IQR)	7 (3.0-14.0)	4 (2.0-11.0)	1.01 (0.99-1.03)	0.25	NI	NI

[a] The following variables were included in the multivariable analysis: CCI, geographical region, country income level, direct interhospital transfer, ICU treatment abroad and antibiotic use during screening.
[b] Eliminated before the final step in backward selection.
[c] Length of hospital stay data was available for 267 patients, of whom 82 (30.7%) were MDRO colonized.

Abbreviations: AOR, adjusted odds ratio; CI, confidence interval; ICU, intensive care unit; IQR, interquartile range; NA, not applicable; NI, not included; OR, odds ratio; Ref, reference.

Alt text: Supplementary table 3 is summarizing the study population's demographic characteristics and the results of univariate and multivariate risk factor analyses for MRSA colonization. Risk factors for MRSA colonization; increasing Charlson Comorbidity Index and decreasing country income level

Supplementary table 4 Annual proportions of MDRO- and ESBL-PE-colonized patients among children hospitalized abroad and screened in Helsinki University hospital, January 2010 – December 2024.

Screening year	No. of patients per year, n	MDRO + per year, n (%)[a]	ESBL-PE + per year, n (%)[b]
2010	7	1 (14.3)	0 (0.0)
2011	36	11 (30.6)	9 (25.0)
2012	32	11 (34.4)	12 (37.5)
2013	47	14 (29.8)	12 (25.5)
2014	34	10 (29.4)	9 (26.5)
2015	35	11 (31.4)	9 (25.7)
2016	39	12 (30.8)	12 (30.8)
2017	24	5 (20.8)	5 (20.8)
2018	31	11 (35.5)	11 (35.5)
2019	49	17 (34.7)	13 (26.5)
2020	18	4 (22.2)	4 (22.2)
2021	19	8 (42.1)	6 (31.6)
2022	25	11 (44.0)	7 (28.8)
2023	29	12 (41.4)	9 (31.0)
2024	34	18 (52.9)	15 (44.1)

[a] Univariate binary logistic regression for trend over time: p=0.03, OR 1.1 (CI95% 1.0-1.1)
[b] Univariate binary logistic regression for trend over time: p=0.16, OR 1.0 (CI95% 1.0-1.1)

Alt text: Supplementary table 4 shows the number of patients screened and found positive for MDRO and ESBL, listed by screening year. An increase in the proportion of MDRO-positive patients can be seen over the years.

Supplementary table 5 Countries[a] and regions of hospitalization and the proportion colonized with multidrug-resistant organisms (MDROs) among children hospitalized abroad, January 2010 – December 2024.

	**n**	**MDRO + n (%)**
**Europe**	**274**	50 (18.2)
Estonia	89	8 (8.0)
Spain	24	1 (4.4)
Russia	18	5 (27.8)
Greece	17	2 (11.8)
Germany	16	3 (18.8)
Italy	10	2 (20.0)
Ukraine	10	5 (50.0)
France	9	1 (11.1)
Kosovo	8	4 (50.0)
Sweden	8	2 (25.0)
UK	8	0 (0)
Latvia	6	2 (33.3)
Austria	5	2 (40.0)
Bulgaria	5	2 (40.0)
Belgium	4	0 (0)
Croatia	4	0 (0)
Norway	4	1 (25.0)
Poland	4	1 25.0)
Romania	4	2 (50.0)
Cyprus	3	1 (33.3)
Netherlands	3	0 (0)
Albania	3	2 (66.7)
Republic of Macedonia	2	2 (100)
Switzerland	2	0 (0)
Other	8	2 (25.0)
**Asia**	**68**	40 (58.8)
Thailand	20	5 (25.0)
India	14	12 (85.7)
Pakistan	8	8 (100)
China	7	5 (71.4)
Nepal	4	2 (50.0)
Philippines	3	1 (33.3)
Vietnam	3	2 (66.7)
Japan	2	1 (50.0)

Malaysia	2	1 (50.0)
Other	5	3 (100)
**North Africa and Middle East**	**60**	33 (55.0)
Turkey	25	11 (44.0)
Iraq	8	6 (75.0)
Egypt	6	6 (100)
Marocco	5	1 (20.0)
Jordan	4	4 (100)
Algeria	3	1 (33.3)
Iran	3	2 (66.7)
United Arab Emirates	3	1 (33.3)
Other	3	1 (33.3)
**Sub-Saharan Africa**	**41**	32 (78.0)
Somalia	12	12 (100)
Ethiopia	7	6 (85.7)
Nigeria	5	5 (100)
Cameroon	4	2 (50)
Kenya	3	3 (100)
South Africa	3	1 (33.3)
Republic of the Congo	2	2 (100)
Other	5	1 (20.0)
**North America**	**8**	0 (0)
USA	6	0 (0)
Canada	2	0 (0)
**Latin America, Caribbean islands**	**7**	3 (43.9)
Colombia	3	1 (50.0)
Other	4	2 (50.0)
**Oceania**	**1**	0 (0)
Other	1	0 (0)

[a] Countries with at least two hospitalized patients are shown, the rest as others
[b] Two patients were hospitalized in two different countries in Asia
[c] Two patients were hospitalized in two different countries in Sub-Saharan Africa

Alt text: Supplementary table 5 shows a list of hospitalization countries with most patients hospitalized in Europe (274 of 459). The proportion of MDRO-colonized patients varied across regions, reflecting the average percentage per region.
